# The Roberts-Hawley Goat Lymph Compound

**Published:** 1901-08

**Authors:** W. P. King

**Affiliations:** San Antonio, Texas


### TEXAS MEDICAL JOURNAL.

ESTABLISHED JULY, 1885.

PUBLISHED MONTHLY.—SUBSCRIPTION $1.00 A YEAR.

VOL. XVII. AUSTIN, AUGUST, 1901. No. 2.

### Original Contributions.

For Texas Medical Journal.

### The Roberts-Hawley Goat Lymph Compound.

W. P. KING, M. D., SAN ANTONIO, TEXAS.*

The readers of the TEXAS MEDICAL JOURNAL (Daniel's "Red Back") will remember my article with the above title in the March number. I do not know how the facts stated in that article strike the average practitioner. I know that before I used this compound and accomplished what I have with it, and before I was acquainted with the facts of the experience of other practitioners with it, I would have received such a statement with a grain of salt, and it may be that I would have wanted to know all about the author before accepting his statements as being gospel truth. I do not, therefore, fall out with those who have not known me for a lifetime when they doubt; for, from the daily experiences of the average practitioner, such facts do appear incredible. He cannot see nor understand how a compound of this kind can accomplish the results claimed for it in diseases and ailments ordinarily intractable to the influence of other remedies, and even to remedies which, from their known therapeutic action, seem to be applicable to such diseases. I feel quite sure that had the claims for this remedy been made to me, that are made, by any other than some well known friend of undoubted integrity, I would have scouted the whole thing as being unbelievable. Therefore, I do not lose my temper and order "pistols and coffee for two" when some doctor

[*Ex-Vice-President American Medical Association; ex-President Missouri State Medical Association; ex-Chief Surgeon Missouri Pacific Ry. Co.; President American Animal Therapy Association.—EDITOR.]

says, "Well, doctor, don't you think that this improvement came from some remedy that the patient had been taking before?"

Such propositions, in view of my own experience, are a little annoying; but human nature is a queer thing. Thomas had to put his hand in the wound in the Savior's side before he would believe that He was the veritable "Son of Man." Therefore, I am disposed to be charitable, and am willing to "wait patiently about," like Mary's little lamb, until each doctor shall have experience of his own, as I did, until he is convinced, and begins to shout and proclaim the good news to other physicians. He needs no further "missionary work" in his behalf, after he has once had a favorable case in which to try it.

I wish to repeat here, what I have often said before, that I believe this remedy to be the greatest discovery, the greatest boon to mankind that has been discovered since the civilization of our race. I believe that it has cured more cases and is destined to cure more cases of the heretofore incurable kind,—tuberculosis of the lungs, and of the lymphatic glands, chronic rheumatism, diabetes, Bright's disease, hemiplegia, locomotor ataxia, fatty infiltration of the heart, and myocarditis with arterio-sclerosis, epilepsy, etc., than all the other remedies known to the profession.

Mentioning epilepsy above, brings to my mind the fact that I have not heretofore laid as much stress on the action of the lymph in this disease as I should. I presume that it is because, with all my large and varied experience, I have not, as yet, treated a case with the lymph, and yet, from what I see of reports from other physicians, the results in the treatment of this disease are simply marvelous. This disease, of all diseases, marks the afflicted one as an open grave in the household where he or she belongs; and, of all diseases, it is the one disease which parents and friends will make almost any sacrifice in order to relieve the afflicted one. A letter from my former office partner in Kansas City (Dr. Geo. B. Norberg) which reaches me, as I write this, tells me of a case of a man suffering from epilepsy in its very worst form, which had been brought to him by a doctor friend of ours in Kansas (Dr. Walthall, of Paola) for consultation.

On Dr. Norberg's advice, Dr. Walthall returned home with the patient and gave him the lymph treatment. Dr. Norberg says, in his letter to me, "This patient walked into my office a few days ago in order to tell me that he was perfectly well, and had not had a seizure for several months." From what I can learn from reports of physicians who have treated cases of epilepsy, this is the usual result in the treatment of this terrible disease. In view of these facts, and in view of the awful havoc which this disease makes of the brain and entire nervous system of the afflicted one, ought any physician to hesitate a moment?

I will report the following additional cases, treated by myself and others, since I wrote my paper for the March number of the JOURNAL:

CASE 1. PULMONARY TUBERCULOSIS.—A. D. McG., age 48, Alto, Texas. Farmer.

This patient came to me early in April. The exertion of traveling all night and getting to my office from the railroad train, so prostrated him, that he almost collapsed on entering my office. After he had rested and recovered, I made a careful examination. I found tubercular infiltration of almost the entire left lung and the apex of the right, with a large cavity in the left. He had been affected with tuberculosis for over a year, and was extremely emaciated and weak. After a careful examination I told him frankly that I did not believe anything would cure him, and I did not think it worth while for him to go to the trouble and expense to take the remedy, as I had a serious doubt whether it would do him any good at all. He seemed much disappointed. He had come a long way and had built hopes on being benefited by this remedy, and he begged that a trial be made in his case any way. I consented, and put him under the usual preparation. After he began to take the remedy, he declared that he felt better after he had taken the second dose. The lymph acted almost like magic in this case, and the patient grew stronger, breathed better and slept better from day to day, so that, while he could not walk fifty yards when he came to me, within ten days I am sure he could have gone out and walked a mile. It was simply a triumphal procession from day to day, and after a few days treatment, instead of coughing from one to two hours in the morning, in order to clear out his lungs, the expectorations were so free and easy that he would clear them in ten minutes. He took two treatments of thirty-five days each. He gained in weight and strength, and his appetite improved, so that he was quite comfortable and happy when he left me, in the early part of June. Examination showed that the lungs had almost entirely cleared up, and he was expectorating very little, and that little came up without any trouble at all. I should have noted in the proper place that this patient was taking two to three grains of morphine daily when he came to me, which he voluntarily quit as he grew stronger.

CASE 2. SPASMODIC ASTHMA.—D. Lawyer, age 62. Shelbyville, Mo.

This patient, who had been away from his home for quite a long time on account of severe and repeated attacks of spasmodic asthma, was treated by Dr. C. M. Decker, of this city. He was much emaciated, weak and worn out from his long struggle with the asthma. Within about two weeks after beginning treatment, he ceased to have any further attacks. He took only four drachms of the lymph, and then decided to return to his home in Missouri. He took with him another fourth treatment, and informed me that he would send for and take a whole treatment as soon as he arrived home. He improved so much in weight, strength, and in his personal appearance, that he scarcely looked like the same person.

CASE 3. SPASMODIC ASTHMA.— Wm. Mullenix, age 38, farmer. Alto, Texas.

This patient was induced to come to me by Mr. McG. (Case 1). The history of this case showed him to have been a great sufferer. For five months he had struggled in the grasp of this terribly exhausting malady, and had had scarcely any relief, excepting the little obtained by burning paper saturated with nitrate of potash and strammonium. He was considerably emaciated, had a poor appetite, very little strength. He never had an attack after he took the first injection of eight drops of lymph. He gained fifteen pounds in three weeks, and was altogether made a very happy man. He took less than one full treatment. If this patient continues to reside in the same locality, in the timber in Cherokee county, I fear that he may have a return of the asthma. So I have advised him that, if it be possible, he should move to another locality.

Case 4. Fatty Infiltration of Heart—Arterio-Sclerosis. *Dr. J. J. Pitts, age 48. Lone Grove, Texas.

This case has a most remarkable history, and stands today as a living monument to the value of this new animal remedy. Dr. Pitts belongs to a tuberculous family, most of his near relatives having died from that disease, and he was himself pronounced to be dying from tuberculosis when a young man, but fully recovered. At twelve years of age he was paralyzed in both lower extremities; but, notwithstanding this, he studied medicine and has practiced his profession for about a quarter of a century. In recent years he has been growing enormously fat, and, although his lower extremities were undeveloped (he having gone on crutches since he was paralyzed), his body weight went up to 287 pounds. With this enormous increase of fat there was, no doubt, an infiltration of fat in the muscular interspaces of the heart, as he soon began to show symptoms of heart degeneration, which after a while developed into real attacks of angina pectoris. These grew worse from time to time, until last November, when he had a serious attack which came near taking his life. From all the evidences in the case, I am prepared to say that there was the usual dilatation of left ventricle and accompaning arterio sclerosis. A neighboring physician attended him, and the doctor grew so very ill that the attending physician, after seeing him on January 8th, went home and wrote him a letter, advising that he arrange his affairs and prepare for death. In the same mail he received one of my circular letters regarding the lymph. As a dying man he was, of course, impressed by my letter, and he wrote me at once, but notwithstanding I gave him some encouragement in my reply, he wrote me that he was going over to Comanche and die amongst his friends. Arriving there he found that Dr. McCarty, whom he went to consult, had just sent for two treatments. Dr. McCarty persuaded Dr. Pitts to take the remedy. In a little over two weeks Dr. Pitts lost thirty pounds under the treatment. He then felt so well that he returned home, sent for a treatment, and has continued to treat himself ever since. He has now taken nearly three treatments. He writes me

*Name used by permission.

that he has lost, altogether, sixty pounds and is still throwing off fat at the rate of three pounds per week. Every symptom of his heart trouble ceased long ago, and he reports to me that examination reveals the fact that his heart action is perfectly normal. He eats well, sleeps like a baby, and is, of course, a very happy man, as we all are when we feel that we have been practically snatched from the mouth of the grave.

CASE 5. NEURASTHENIA.—Mrs. R., age 30. Missouri.

This patient underwent a series of surgical operations and procedures early in the year; first a curettement and afterwards the extirpation of a suppurating vulvo-vaginal gland. She was, I learn, exceedingly sensitive at the beginning, and each succeeding chloroforming seemed to render her more and more so; even after the first operation she worried and wept a great deal and could not sleep. She would not permit the wound to be touched without being first anæsthetized, and each time after taking any chloroform she exhibited strong evidences of a general neurasthenia. When she came to me she was sleeping very little, and worrying a great deal about matters that amounted to practically nothing. She did not eat, and was altogether seriously ill. She was put upon the lymph treatment at once, and after a few days she began to grow better, so that now, after about six weeks treatment, I regard her as free from neurasthenia though still pale and weak and nothing like as strong as she was before she became ill. There is no doubt in my mind that a condition of cerebral anemia was brought on by the repeated anæsthetizing, and this, added to an already existing spinal neurasthenia, did the mischief.

CASE 6. SEQUELAE OF PLEURITIS WITH EFFUSION.—Mrs. F., age 38. Missouri.

This lady suffered in the latter part of last year from an attack of pleuro-pneumonia. I am inclined to believe, however, that the pleurisy part of it was overlooked. When she came to me, I found that there had been an effusion of serum in the left pleural cavity which had collapsed the lung, but the effusion had been absorbed when I first saw her. There was very little air entering that lung, and the chest movement on that side amounted to nothing at all when she breathed. She coughed almost incessantly day and night. I put her upon the treatment, and trusted to the cellular action of the remedy to restore the lung to its normal capacity. She improved from the first, and in about a week came in one morning boasting that she had not coughed any the night before; and, upon examination, I found that the lung almost wholly expanded throughout. I presume every physician has had those cases in which a lung had been collapsed from an effusion and afterwards gradually expanded after the pressure of the water around it had been absorbed; but I confess that this is my first experience in witnessing a lung expand in one night, and I am bound to attribute the fact to the rapid cell restorative action of the lymph. This patient was pale and weak from her long sickness when she came to me; but she gained eight pounds within a few weeks, and returned home a few days ago, in perfect health and strength, and looking as rosy as a young girl.

CASE 7. PULMONARY TUBERCULOSIS WITH CHRONIC INTERSTITIAL NEPHRITIS.—Mrs. E., age 57. Waxahachie, Texas.

This lady came to me early in March. Examination revealed general tubercular infiltration of left lung with cavity, and infiltration at apex of right. Her feet, hands and face all showed dropsical effusion. I would perhaps have attributed this to the general weakness of the circulation and tissues, had she not informed me that her physician had found large quantities of albumen and other evidences of "Bright's" disease in her urine. She responded beautifully to the lymph treatment, and within four weeks after she came to me, the lungs were practically free from infiltration. She was extremely weak when she came to me, and coughed almost day and night. She ceased to cough at the end of the fourth week, but would spit up great quantities which seemed to arise in her throat without the effort of coughing. She slept well, but never regained her appetite. I should have said she had suffered greatly from dyspepsia. There was an occasional recession of the general dropsical condition, but it would reappear within a day or two. I soon became convinced that while her tubercular condition was relieved, there were structural changes of the kidneys to such an extent that to relieve the interstitial nephritis was impossible. After about ten weeks treatment, she began to show signs of uremic poisoning, which gradually increased until she finally passsed into a condition of profound coma and died. This was a case in which, had the patient been suffering from tuberculosis alone, I feel quite sure I would have sent her home well; but she suffered from too many destructive diseases, and at her age, to cure her was impossible.

CASE 8. CHRONIC BRONCHITIS.—Mrs. W., age 34. Texas.

This lady came to me in the latter part of the month of March. She had suffered for many years from bronchitis. She coughed almost incessantly, the exertion of which had almost worn her down to a skeleton. She ate but little, slept poorly, and was extremely nervous. There were the usual findings in her lungs. On a general examination of her case, I found that she had laceration through the posterior lip of the cervix uteri. After giving her the lymph for two weeks, during which time she improved in strength, appetite and ability to sleep, I then repaired the laceration, after which I kept her in bed for two weeks, during which time I gave her the lymph twice a day. By the time she got out of bed from the operation, her bronchitis was practically well. She continued to take the treatment, however, for a few weeks afterwards, when she returned home, perfectly cured. She has returned here once on a visit since, and is still well, as she eats and sleeps normally, and does not cough at all.

CASE 9. RHEUMATOID GOUT.—Mrs. K., Cincinnati, Ohio; age 40; stenographer.

This was a badly afflicted patient. She had suffered from above condition for many years. There were exostoses across the epiphyses of the phalanges of both fingers and toes. She suffered greatly nights and mornings. Her toes got into a state of, what she called, "erection"—strutted, stiff and immovable each morning, and, at such times, she cried and screamed with pain.

She had been treated for years by a rigid diet regimen and the so-called anti-rheumatic remedies.

I gave her the lymph in doses, beginning with six drops, and gradually ran up to ten and twelve drops. Within four weeks all pain had ceased, except a severe pain (neuralgic in character) in the toe next the little toe on each foot. After careful examination I became satisfied that this was not a gouty pain, but that it was a case, so far as it pertained to those two toes, of "Morton's painful toe." I disarticulated both toes at the metatarso-phalangeal articulation and removed more than one inch of the metatarsal bone.

When she recovered from this operation, all pain had ceased. She took treatment only about six weeks. In this time the exostoses were greatly reduced in size, and all pain and tenderness left both her feet and hands. She was well and free from pain, and having recovered the use and flexibility of her fingers so as to use the typewriter, and feeling obliged to resume her work as soon as she could do so, she returned home and resumed her position.

She knows what to do, however, in case she has future trouble. No one can persuade her to even discuss any other remedy than the lymph. The marked benefits were so rapid and so plain, after everything else had failed, that she cannot but be enthusiastic.

I have received and am receiving reports from a good many doctors in Texas and Old Mexico, reporting cases under treatment, and these reports are most favorable in most of the cases; yet as the lymph has not been used in Texas, outside of San Antonio and one or two other places, except for a short time, most all cases that are being treated by other doctors are yet under treatment. In many of the cases the reports are not full, and I will save report on such cases for another paper. I have a number of other cases of general and local neurasthenia which I have treated and cured, but will let the one case reported suffice. I have treated a number of other cases of pulmonary tuberculosis in which cases the patients are practically well, and I have had failure in a few cases in which the patients were suffering from secondary mixed infection and in which the pyogenic fever was running high in the afternoon and dropped below normal in the morning, with exhausting night sweats, hectic flushes, and all other evidences of pus poisoning. Most of the latter cases were greatly benefited in the start, and were rendered much more comfortable on account of easier expectoration and refreshing sleep at night, but they succumbed finally to the ravages of this terrible disease. In this connection I wish to call attention to the letter of Dr. Julius Ullman, of Buffalo, New York, in the *Journal of the American Medical Association*, May 4th, 1901, in which he recommends brewer's yeast as a remedy in secondary mixed infections of tuberculosis. It is claimed that the nucleinic acid formed by the fermentation of the yeast, is destructive to pus germs and their products, and that it therefore lowers the temperature by destroying the cause of the high range of temperature. I have used the yeast in connection with the lymph in several cases, and in two cases I am sure I got decided benefits from its administration. The yeast must be fresh and kept cold and two to four ounces given every three or four hours, or in double this quantity by enema, when it can not be borne by the stomach. I thought it important that doctors who are treating cases of pulmonary tuberculosis with the Roberts-Hawley Goat Lymph Compound should know of this brewer's yeast as an adjuvant.

I have been using the lymph by intra-spinal injection in cases of locomotor ataxia. Reports from Dr. Hamilton Forline, of Chicago, show that it produces much greater and more rapid benefits when administered in this way than when given in the usual way. It should not be given oftener than two or three times a week, and the physician should never give more than four drops to begin with and gradually increase the dose. Dr. Forline has had a needle and syringe made for this particular purpose, which I find to be all that is needed in this operation. The needle is introduced between the second and third or third and fourth lumbar vertebræ, until the point of the needle enters the covering of the cord. This is indicated by a flow of the spinal fluid. The lymph which has been diluted with an equal quantity of sterile distilled water is injected after as many drops of spinal fluid have escaped as you intend to inject of lymph and water. The needle is then withdrawn and the thumb held over the puncture for about a half minute and the place is then sealed with collodion and cotton. In a few cases of locomotor ataxia the sufferings of the patient are quite severe, after these injections, for twenty-four to forty-eight hours, but usually after the suffering is over the patient shows great improvement in the way of a lessening of the lightning pains, and improved co-ordination. In the June *Journal of the American Animal Therapy Association* Dr. Hawley says he is now preparing a lymph for intraspinal injection which he has deprived of the elements that produce pain in locomotor ataxia. The cases in which I have been using it are very unfavorable, one of them being a case of mixed ataxia and spastic paralysis, another associated with long continued neurasthenia with profound deafness and great irritability of the nervous system, and the other having melancholia with delusions, and yet, although I have used it only five times in one case, twice in another and once in the other, I am sure that there has been marked improvement in each case.

I desire to call the attention of the readers of the Journal especially to the value of this remedy in certain diseases of the nerves of special sense, more particularly to diseases of the optic and auditory nerves (which result in blindness and deafness), and in certain forms of insanity.

Dr. Joseph R. Hawley, of Chicago, in Bulletin No. 39, May 22, 1901, says:

"Dr. Hamilton Forline's unusual result in a case of optic atrophy with total blindness will be described in detail in next journal. As this case is exciting a great deal of interest among members of the profession, it is cited briefly in this report. The value of this result, as a proof of efficacy of our cell tonic, is greatly enhanced by the fact that several widely known oculists had previously confirmed diagnosis beyond question, and made absolutely hopeless prognoses: Male, age 40; optic atrophy from traumatic injury of one eyeball, degeneration having rapidly extended to nerve of other eye. Totally blind. Treated two months by ordinary hypodermatic method. Now able to walk through business part of city, to distinguish objects several blocks distant and to read large print."

It seems to me that this is a matter of vital importance to the profession, and especially so to persons about to become blind from optic atrophy. The same may be said regarding those about to become deaf from disease of the auditory nerve. If such results can be accomplished after a person has become totally blind, what may be expected if those cases are properly treated in the beginning?

In a case of lumbago and sciatica, treated by myself, in which the patient (a man 82 years old) was almost totally deaf, the patient recovered his hearing to a marked degree after six weeks' treatment. The lumbago and sciatica disappeared after two weeks.

Regarding the treatment of cases of insanity, Dr. Albert A. Lowenthal, of Chicago, a specialist in neurology, says, in the February number of the *Journal of the American Animal Therapy Association,* speaking of the lymph treatment:

"In the past two years I have taken fifty-six patients of mental disease out of State institutions and private hospitals for the insane, and in no instance have any of these patients had to return to these or other institutions or hospitals."

It does seem to me that this is a matter of vast moment to those having members of their families in insane asylums. There is no doubt in my mind but what there are numbers of cases in all hospitals or asylums for the insane whose cases would prove amenable to this treatment, if it were only used in such cases.

### ADDENDUM.

I wish to advise those who wish to get something really valuable on the subject of the new animal therapy to send to Dr. J. R. Hawley, Medical Director, 3421 South Park Ave., Chicago, Illinois, and get the June number of the *Journal of the American Animal Therapy Association.* I don't hesitate to say that I believe it contains more of real, valuable interest to the profession than any other journal ever published in the English language.

